# Reaching the highest efficiency of spin Hall effect of light in the near-infrared using all-dielectric metasurfaces

**DOI:** 10.1038/s41467-022-29771-x

**Published:** 2022-04-19

**Authors:** Minkyung Kim, Dasol Lee, Younghwan Yang, Yeseul Kim, Junsuk Rho

**Affiliations:** 1 Department of Mechanical Engineering, Pohang University of Science and Technology (POSTECH), Pohang, 37673 Republic of Korea; 2 Department of Biomedical Engineering, Yonsei University, Wonju, 26493 Republic of Korea; 3 Department of Chemical Engineering, Pohang University of Science and Technology (POSTECH), Pohang, 37673 Republic of Korea; 4 POSCO-POSTECH-RIST Convergence Research Center for Flat Optics and Metaphotonics, Pohang, 37673 Republic of Korea

**Keywords:** Metamaterials, Sub-wavelength optics, Characterization and analytical techniques

## Abstract

The spin Hall effect of light refers to a spin-dependent transverse splitting of light at a planar interface. Previous demonstrations to enhance the splitting have suffered from exceedingly low efficiency. Achievements of the large splitting with high efficiency have been reported in the microwave, but those in the optical regime remain elusive. Here, an approach to attain the large splitting with high efficiency in the near-infrared is proposed and experimentally demonstrated at 800 nm by using a dielectric metasurface. Modulation of the complex transmission of the metasurface leads to the shifts that reach 10*λ* along with efficiencies over 70% under two linear polarizations. Our work extends the recent attempts to achieve the large and efficient spin Hall effect of light, which have been limited only to the microwave, to the optical regime.

## Abstract (web summary)

Here, Junsuk Rho and co-workers propose and experimentally demonstrate a metasurface supporting a large and efficient spin Hall effect of light in optical wavelength. The spin Hall shifts reaching ten wavelengths with efficiencies over 70% are observed.

## Introduction

Metasurfaces, nanostructure arrays that are designed to exhibit desired optical responses, have emerged as powerful tools to modulate various features of electromagnetic waves such as amplitudes, phases, and polarizations^1^. Whereas materials in nature have limited optical properties, the metasurfaces have shown unprecedented light manipulation capabilities despite their deep-subwavelength thickness. To maximize the light–matter interaction in a short spatial path length, the nanostructures that compose the metasurfaces have been constructed from plasmonic materials^2,3^. At optical wavelengths, however, plasmonic metasurfaces generally suffer from low efficiency due to Ohmic loss, thereby hindering their practical implementations. As alternatives, all-dielectric metasurfaces, constituents of which are all made of dielectric materials, have been suggested to manipulate the optical responses efficiently^4–6^. The dielectric metasurfaces have reproduced many exotic optical responses of the plasmonic metasurfaces such as polarization control^5,7^, ultrathin lenses^8–11^, and holographic display^12–15^ with even higher efficiencies than the plasmonic metasurfaces. Furthermore, the metasurfaces’ compactness, compatibility, and ease of integration have made them a viable replacement for bulky optical components^16–19^.

During the past decade, a variety of optical analogies of the spin Hall effect^20,21^ such as spin Hall effect of light (SHEL)^22,23^, optical Magnus effect^24^, spin-dependent angular splitting^25^, plasmonic spin Hall effect^26,27^, and optical spin Hall effect^28^ have regained extensive scientific interest when combined with structured media^29–33^ such as metasurfaces and metamaterials. Among them, the SHEL is a transverse and spin-dependent spatial displacement of light at refraction or reflection^34–36^. At an interface between naturally occurring materials, the SHEL exhibits in general a shift that is much smaller than the wavelength^23^. However, specifically designed metasurfaces and metamaterials can enhance the shift by several orders of magnitude^37–44^ from a few micrometers^45^ to a few hundreds of micrometers^46,47^. Unfortunately, the drastic enhancement of the SHEL generally entails extremely low efficiency, as a result of a vanishing Fresnel coefficient^37,38,40^. A simultaneous enhancement of the shift and efficiency has been achieved very recently in the microwave regime using hyperbolic^48^ and index-near-zero^49^ metamaterials. However, these demonstrations cannot be readily adapted to the optical regime that encompasses ultraviolet, visible, and infrared wavelengths (10 nm < *λ* < 1 mm) because the operating principles rely on effective medium properties that require deep-subwavelength structures going down from hundreds of micrometers to a few tens of nanometers scale for optical operations. The lack of fabrication methods for such complicated structures and high optical losses of metals in the optical regime hinder the realization of the large and efficient SHEL. Thus, a large SHEL with high efficiency has not been achieved in the optical regime.

Here we propose an all-dielectric metasurface to enhance both the shift and efficiency of the SHEL simultaneously in the near-infrared regime. In contrast to the previous approaches that have demanded a three-dimensional or stacked bulky structure to achieve the desired effective medium properties, here, a radically different approach is used by exploiting a single-layer metasurface as a compact and versatile route to modulate the transmission coefficients. To achieve high efficiency, the metasurface is designed to yield high transmission amplitudes for both *s* and *p* polarizations, with the phase difference between the two linear polarizations being far from zero. Modulation of the complex transmission results in a simultaneous enhancement of the shift and efficiency under both horizontal and vertical polarizations at 800 nm. More specifically, we experimentally demonstrate a shift and efficiency that reaches 10.0*λ* and 71%, respectively, under horizontally polarized incidence and 9.9*λ* and 79%, respectively, under vertically polarized incidence. Our work bridges the gap between metasurface research and their implementation towards efficient spin-sensitive devices. The large SHEL with high efficiency based on a dielectric metasurface will find wide applications in ultrahigh precision measurement and compact, spin-sensitive optical devices in nanoscale.

## Results

### Principle

When transmitted at an optical interface, a linearly polarized Gaussian beam undergoes a transverse spatial shift by an amount^23^

1 $$\begin{array}{r}{\delta }_{H}^{\pm }/\lambda =\pm \frac{\cot {\theta }_{i}}{2\pi }\left(1-\frac{| {t}_{s}| }{| {t}_{p}| }\cos \phi \right),\\ {\delta }_{V}^{\pm }/\lambda =\pm \frac{\cot {\theta }_{i}}{2\pi }\left(1-\frac{| {t}_{p}| }{| {t}_{s}| }\cos \phi \right),\end{array}$$

where superscripts + and − represent left- and right-circularly polarized components of the transmitted beam, respectively, subscripts *H* and *V* correspond to the horizontal and vertical incident polarization respectively, *λ* is the wavelength, *θ*_*i*_ is the incident angle, *t*_*s*_ and *t*_*p*_ denote Fresnel coefficients of the *s*- and *p*-polarized incidence respectively, and *ϕ* = arg(*t*_*s*_) − arg(*t*_*p*_) is the phase difference between the two Fresnel coefficients. Note that the Fresnel coefficients and *θ*_*i*_ are those of the central wave vector. Equation (1) is valid under condition of ${(2\pi w/\lambda )}^{2}\gg {\cot }^{2}{\theta }_{i}$ for a beam waist *w*, which is true except for a tightly confined beam under near-normal incidence (*θ*_*i*_ ≪ 1°). This narrow angular regime is where a transition from the SHEL to vortex generation occurs^50^ and is out of our consideration.

**Fig. 1:**
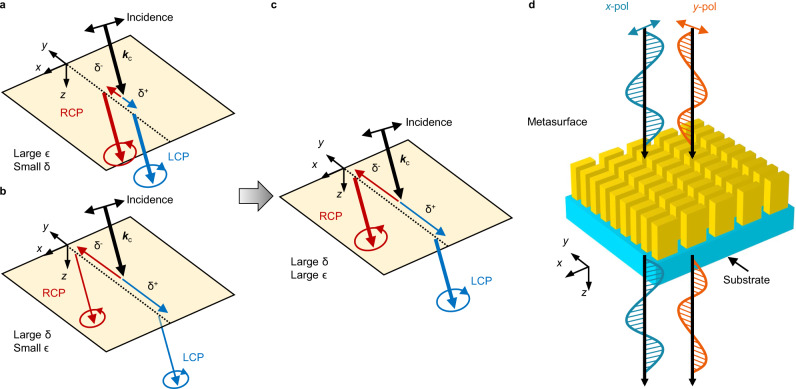
Schematic of the spin Hall effect of light (SHEL) and a dielectric metasurface to enhance the shift *δ* and efficiency *ϵ* of the SHEL simultaneously. **a**, **b** Illustration of the SHEL in previous demonstrations. The width of the transmitted beams denotes their intensities. **a** The efficient SHEL is limited by the small shift, **b** whereas the enhancement of the shift entails a low efficiency. **c** Large SHEL with high efficiency. **d** A metasurface that transmits *x*- and *y*-polarized incidence with unity amplitudes (∣*t*_*x*_∣ = ∣*t*_*y*_∣ = 1) but with the opposite phase (arg(*t*_*x*_) − arg(*t*_*y*_) = ± *π*).

The shift is generally much smaller than the wavelength at an interface between naturally occurring media (Fig. 1a). An intuitive approach to enhance the shift is to reduce the Fresnel coefficients in the denominator (∣*t*_*p*_∣ for horizontal and ∣*t*_*s*_∣ for vertical case); however, given that the efficiency is directly related to the Fresnel coefficients^48^ (*ϵ*_*H*_ = ∣*t*_*p*_∣^2^ and *ϵ*_*V*_ = ∣*t*_*s*_∣^2^, Fig. 1b), the method has the intrinsic limitation that it also degrades the efficiency significantly. Recently, two approaches to achieve large SHEL and high efficiency have been proposed^48,49^ (Fig. 1c). The key idea is to fix the Fresnel coefficient in the denominator to unity to ensure unity efficiency while enhancing the shift by reducing *θ*_*i*_. At an interface between two isotropic media, because the two Fresnel coefficients are degenerate under normal incidence, both $\cos \phi | {t}_{p}| /| {t}_{s}|$ and $\cos \phi | {t}_{s}| /| {t}_{p}|$ are close to unity at a small *θ*_*i*_, thereby giving rise to a subwavelength shift. To attain a large shift, an anisotropic medium^48^ and near-index-zero metamaterial^49^ have been proposed to differentiate the amplitude and phase of the remaining Fresnel coefficients, respectively. These previous methods have been, however, demonstrated in the microwave regime where metals behave as perfect electrical conductors. Realization of the large SHEL with high efficiency in the optical frequencies using this method is not trivial due to the high optical losses of metals and challenging fabrications and measurements.

A dielectric metasurface can be used to overcome these difficulties, because of its low loss and the ability to control its complex transmission. Nanostructure arrays that have dimensions carefully chosen to modulate the amplitudes and phase of the transmitted beam can result in a large SHEL and high efficiency simultaneously. Ideally, a metasurface that transmits both *x*- and *y*-polarized incidence with unity amplitudes but with a phase difference of *π* or −*π* yields a SHEL that reaches $\delta^\pm / \lambda = \pm\!\!\cot {\theta }_{i}/\pi$ with near-unity efficiency at a small *θ*_*i*_ ( ≪ *π*/2) (Fig. 1d). In short, the design rule is to build a dielectric metasurface operating as a half-wave plate (∣*t*_*s*_∣ = ∣*t*_*p*_∣ = 1, *ϕ* = ± *π*) under normal incidence and to examine the SHEL at small *θ*_*i*_. Whereas the capabilities of metasurfaces to manipulate the transmission or reflection have been well known^5,7,51^, no attempt has been made to use these qualities to produce a large and efficient SHEL. Furthermore, in comparison to the commercially available half-wave plates, this metasurface-based approach has advantages of compactness and compatibility.

### Design and fabrication

**Fig. 2:**
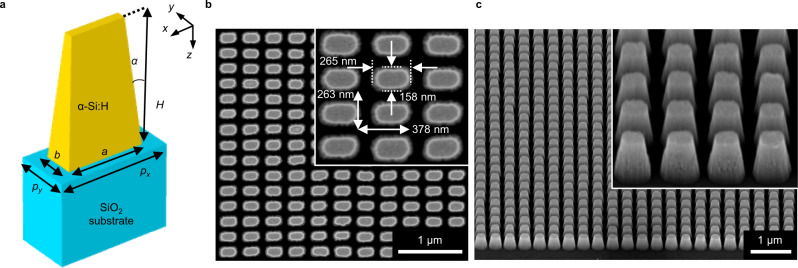
Design of a dielectric metasurface. **a** Schematic of a unit cell. Geometric parameters are given as: height *H* = 400 nm, length *a* = 265 nm, width *b* = 158 nm, periodicities *p*_*x*_ = 378 nm and *p*_*y*_ = 263 nm, and taper angle *α*. **b**, **c** Scanning electron microscopy images of the fabricated sample. **b** Top view, **c** perspective view. Inset shows a magnified image.

To ensure the phase difference between *t*_*s*_ and *t*_*p*_, a unit design of the metasurface has an anisotropic geometry of a rectangular-shaped nanorod on a fused silica substrate (Fig. 2a). This rectangular nanorod is known to induce a Pancharatnam–Berry phase^25,52^ under normal incidence. However, the phase contributes negligibly to the SHEL because the Pancharatnam-Berry phase is more dominant than the spin-redirection phase only under normal incidence and at a close vicinity of it^50^ (*θ*_*i*_ ≪ 1°), which is not where we are interested in. Hydrogenated amorphous silicon (a-Si:H)^53^ is used for its high refractive index and low optical losses (Supplementary Note 7). This dielectric nanorod can modulate amplitudes and phases of the incoming light with substantially higher efficiency compared to plasmonic resonators. To find an optimal design for ∣*t*_*s*_∣ = ∣*t*_*p*_∣ = 1 and *ϕ* = ± *π*, five geometrical parameters, height (*H*), length (*a*), and width (*b*) of the nanorod and periodicities (*p*_*x*_ and *p*_*y*_), are determined using a particle swarm optimization^54^ to minimize 2 − ∣*t*_*s*_ − *t*_*p*_∣ at 800 nm wavelength for a taper angle *α* = 5° (see “Methods” section for details). Then the metasurface is fabricated using electron beam lithography (see “Methods” section).

**Fig. 3:**
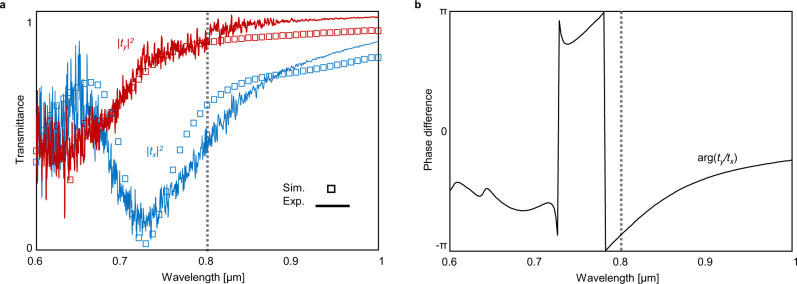
Transmittance of the dielectric metasurface under normal incidence. **a** Simulated (markers) and measured (solid lines) transmittance under normal incidence. **b** Phase difference *ϕ* between the two polarizations obtained by numerical simulation.

The transmittance of the metasurface calculated by using full-wave simulation (see “Methods” section for detailed simulation) and measured by Fourier-transform infrared spectroscopy are shown in Fig. 3a. The simulated and measured transmittance agrees well with each other. A low transmittance at 726 nm under *x*-polarized incidence (Fig. 3a, blue) is attributed to the magnetic dipole resonance manifested by the displacement currents circulating the dielectric nanorods (see Supplementary Note 3). In contrast, the *y*-polarized incidence induces the magnetic dipole resonance at a smaller wavelength near 675 nm because of the smaller rod length and periodicity and thus has a high transmittance at *λ* > 700 nm (Fig. 3a, red). The phase difference *ϕ* varies rapidly near the magnetic dipole resonances and has a value of −0.87*π* at 800 nm (Fig. 3b). To summarize, the metasurface has a high transmittance under both *x*- and *y*-polarized incidences and large *ϕ* at 800 nm, which is advantageous in realizing a large SHEL with high efficiency. Simulations indicate that a metasurface composed of nontapered cells with different geometrical parameters can have *ϕ* = ± *π*, which produces further enhanced SHEL (see Supplementary Note 1).

### Experimental demonstration

**Fig. 4:**
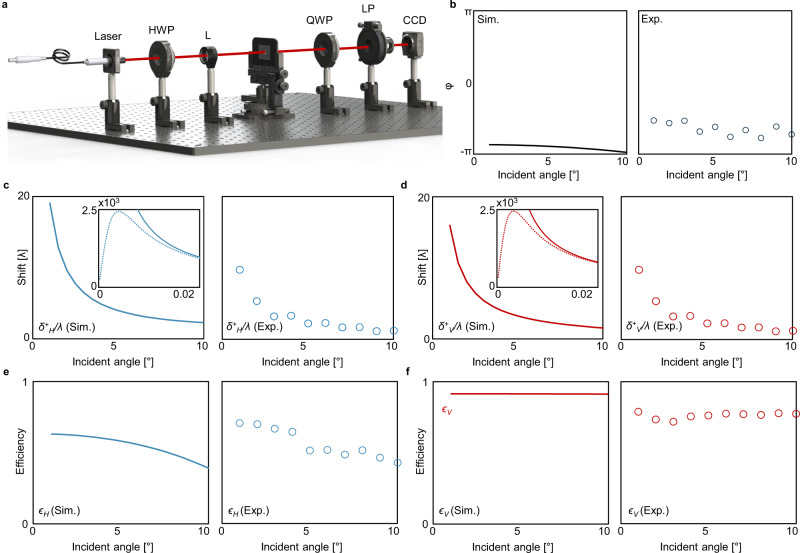
Measurement of the SHEL at 800 nm. **a** Schematic of Stokes polarimetry set-up. HWP: half-wave plate, L: lens, QWP: quarter-wave plate, LP: linear polarizer, CCD: charge-coupled device camera. **b** Phase difference between *t*_*s*_ and *t*_*p*_, **c **${\delta }_{H}^{+}/\lambda$, **d **${\delta }_{V}^{+}/\lambda$, **e ***ϵ*_*H*_, and **f ***ϵ*_*V*_. Curve: simulated, markers: measured. Insets in **c**, **d** show the magnified view for near-normal incidence where the analytic formula (solid) given by Eq. (1) deviates from the exact shifts (dashed) due to the breakdown of the assumption, i.e., ${(2\pi w/\lambda )}^{2}\gg {\cot }^{2}{\theta }_{i}$. Error bars of the measured data are not shown because they are all smaller than the markers.

In general, the shift can be experimentally obtained via either weak measurement^23^ or Stokes polarimetry measurement^55,56^. The former amplifies the shift by several orders of magnitudes where the amplification factor is determined by the setup parameters and the latter measures the Fresnel coefficients and their phase difference under oblique incidence so that the shift can be deduced from Eq. (1). Here the large SHEL with high efficiency at 800 nm is experimentally verified using a Stokes polarimetry setup (Fig. 4a, see “Methods” section for measurement details). The measurements are performed in two ways: first by setting the incident polarization as 45°-linearly polarized one to measure the phase difference *ϕ* and then by setting the incident polarization as horizontal and vertical to measure the amplitudes ∣*t*_*p*_∣ and ∣*t*_*s*_∣ respectively. Then the shifts can be obtained by substituting the measured *ϕ*, ∣*t*_*s*_∣, and ∣*t*_*p*_∣ into Eq. (1). In all measurements, the metasurface is placed tilted by *θ*_*i*_ ranging from 1° to 10° to examine the SHEL at various *θ*_*i*_.

In the first measurement, we obtain the second and third Stokes parameters,

2 $$\begin{array}{rc}{s}_{\!2}&=I({0}^{\circ },4{5}^{\circ })-I({0}^{\circ },13{5}^{\circ }),\\ {s}_{3}&=I(9{0}^{\circ },4{5}^{\circ })-I(9{0}^{\circ },13{5}^{\circ })\end{array}$$

where *I*(*β*, *γ*) is the spatial integration of the intensity when retardation angle of the quarter-wave plate is *β* and rotation angle of the linear polarizer is *γ* (Fig. 4a). Then the measured Stokes parameters are used to calculate^56^ *ϕ* by using a relation of $\tan \phi ={s}_{3}/{s}_{2}$. The measured *ϕ* deviates slightly from the simulated values because of the fabrication imperfections but exhibits a similar tendency (Fig. 4b).

In the second measurement, ∣*t*_*p*_∣ and ∣*t*_*s*_∣ under oblique incidence is obtained by taking the square roots of the intensity of transmitted beam under horizontally (Fig. 4e) and vertically (Fig. 4f) polarized incidence respectively. Note that the intensity is normalized by that measured without the sample. The shifts calculated by substituting the measured *ϕ*, ∣*t*_*s*_∣, and ∣*t*_*p*_∣ in Eq. (1) and their efficiencies are shown in Fig. 4c–f. The shift is enhanced remarkably up to several wavelengths at a small *θ*_*i*_ (≤ 5°) under both horizontal (Fig. 4c) and vertical (Fig. 4d) polarizations. We experimentally observe the shift of 10.0*λ* along with 71% efficiency under horizontal polarization and the shift of 9.9*λ* along with 79% efficiency under vertical polarization at *θ*_*i*_ = 1°. The shifts can be further increased by reducing *θ*_*i*_ except for an extremely narrow region near *θ*_*i*_ = 0°. When *θ*_*i*_ is sufficiently small (≤ 0.01°), Equation (1) deviates from the exact shifts and converges to zero rapidly (Fig. 4c, d, inset). The metasurface exhibits high transmittance for both polarizations even under oblique incidence. Despite the large SHEL, high efficiencies are observed at the wide range of *θ*_*i*_ (Fig. 4e, f). For completeness, the shift is also obtained using the weak measurement technique. The shift is 1.31*λ* under vertically polarized light at *θ*_*i*_ = 10°, which agrees well with the result from the Stokes polarimetry method (Supplementary Note 6). The shift and efficiencies in our work in comparison with previous studies are summarized in Supplementary Note 4.

## Discussion

We experimentally demonstrate simultaneous enhancement of the shift and efficiency of the spin Hall effect of light in the optical regime for the first time by using a dielectric metasurface. Whereas previous approaches to enhance the shift have suffered from low efficiencies and the achievements of a large shift with high efficiency have been limited in the microwave regime, we present the large shift and high efficiency at 800 nm by modulating the complex transmission of the metasurface. Shifts that reach 10*λ* with efficiencies >70% are confirmed experimentally under two linearly polarized incidences. Furthermore, the shift can be further enhanced by reducing the taper angle through an optimization of the etching condition^57^. The simultaneous enhancement of the shift and efficiency can be also achieved in a reflective metasurface by changing geometrical parameters (Supplementary Note 5).

This approach towards the large spin Hall effect of light with high efficiency has two distinct merits. First, it facilitates ultrahigh precision measurements by increasing an amplification factor of a weak measurement. The amplification factor is directly linked to the precision, but its increase is practically bounded by the detectable signal strength. The *n*-fold enhancement of the SHEL efficiency provides *n* times stronger signal, which, in turn, enhances the precision by *n* times. Secondly, the large spin Hall effect of light with high efficiency in the optical regime enables realizing compact photonic devices such as spin-sensitive filters. With these clear advantages, the metasurface-driven simultaneous enhancement of the shift and efficiency will find wide applications in spin-dependent optics.

## Methods

### Numerical simulation and optimization

Transmission coefficients of the dielectric metasurface were calculated using commercial software (COMSOL Multiphysics). For transmission under normal incidence, a unit cell with periodic boundary conditions along the *x*- and *y*-axes and perfectly matched layers and ports along the *z*-axis was simulated. For transmission under oblique incidence, simulation conditions were similar to these, except that the periodic boundary conditions were replaced by the Bloch boundary conditions.

The dielectric metasurface was optimized to minimize 2 − ∣*t*_*s*_ − *t*_*p*_∣ at 800 nm by using particle swarm optimization. Starting from a rectangular nanorod that has height *H*, length *a*, and width *b* and is periodically arranged with periodicities *p*_*x*_ and *p*_*y*_, the five parameters were searched during 100 iterations.

### Sample fabrication

The all-dielectric metasurface was fabricated using standard plasma-enhanced chemical vapor deposition (PECVD), electron-beam lithography (EBL), and reactive-ion etching. The fabrication process begins with the deposition of hydrogenated amorphous silicon (a-Si:H) on a fused silica substrate using PECVD, then spin-coating the a-Si:H with a positive photoresist (Microchem, PMMA A6) at 5000 r.p.m. for 1 min and baking at 180 °C for 5 min. Then a conductive polymer (Showa Denko, Espacer Z300) was spin-coated at 2000 r.p.m. for 1 min to prevent charge accumulation during the subsequent process. The sample was exposed using EBL at an acceleration voltage of 80 kV and developed using a commercial developer (Microchem, MIBK:IPA = 1:3) at 0 °C for 9 min. A Chromium (Cr) mask pattern was deposited using electron beam deposition and transferred to a-Si:H by using reactive-ion etching. Finally, the all-dielectric metasurface was fabricated after Cr mask etching by immersing it in Cr7 etchant for 1 min.

### Stokes polarimetry measurement

A Stokes polarimetric technique was used to measure the shifts of the beam transmitted through the dielectric metasurface. A horizontally polarized source with a specific wavelength of 800 nm and a beam waist of 4 mm was filtered using an acousto-optic tunable filter (AOTF) from a supercontinuum laser (YSL SC-Pro). The polarization state of the incident beam was rotated using a half-wave plate. The incident beam was focused to have a beam size less than the sample size (~500 μm × 500 μm) by a lens and illuminated to the dielectric metasurface at various tilt angles (from 1° to 10°, step: 1°). The beam passed through a quarter-wave plate (Thorlabs, AQWP10M-980) and a Glan–Tompson polarizer (Thorlabs, GL15) in sequence, then was captured by a charge-coupled device camera (Thorlabs, DCC1545M). To minimize errors due to laser fluctuation, ten images were taken sequentially, then averaged to produce one image. Five consecutive measurements were performed in all experiments.

## Supplementary information

Supplementary Information

## Data Availability

The data other than that provided in Source data that support the findings of this study are also available from the corresponding author upon reasonable request. Source data are provided with this paper.

## Source data

Source Data
