# Supplementary material for: Reaching the highest efficiency of spin Hall effect of light in the near-infrared using all-dielectric metasurfaces
Source: Nat Commun. 2022 Apr 19;13:2036. doi: 10.1038/s41467-022-29771-x (PMC9018796; doi:10.1038/s41467-022-29771-x)
Supplement: Supplementary file 1 — Supplementary Information [file 41467_2022_29771_MOESM1_ESM.pdf]

Supplementary Information of
"Reaching the highest efficiency of spin Hall effect of light in the near-infrared using all-dielectric metasurfaces"

Minkyung Kim[1,†], Dasol Lee[1,2,†], Younghwan Yang[1,†], Yeseul Kim[1], and Junsuk Rho[1,3,4*]

[1]Department of Mechanical Engineering, Pohang University of Science and Technology (POSTECH), Pohang 37673, Republic of Korea
[2]Department of Biomedical Engineering, Yonsei University, Wonju 26493, Korea
[3]Department of Chemical Engineering, Pohang University of Science and Technology (POSTECH), Pohang 37673, Republic of Korea
[4]POSCO-POSTECH-RIST Convergence Research Center for Flat Optics and Metaphotonics
[*]jsrho@postech.ac.kr

[†]These authors contributed equally to this work.

Supplementary Note 1. Nontapered metasurface for ideal performance

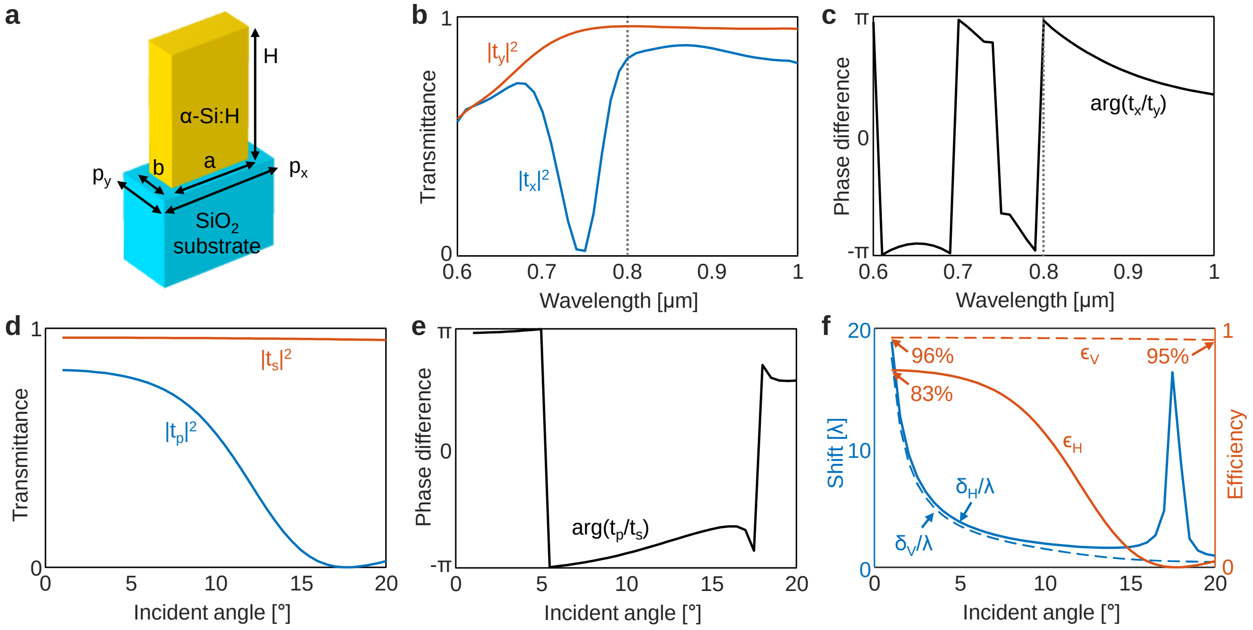

Supplementary Figure 1. Simulation of a nontapered metasurface. (a) Schematic of a unit cell. Geometric parameters are given as: $H = 400$ nm, $a = 250$ nm, $b = 100$ nm, $p_x = 360$ nm, and $p_y = 180$ nm. (b) Transmittance and (c) ϕ under normal incidence. (d) Transmittance and (e) ϕ under oblique incidence. (f) The shift and efficiency under the horizontally (solid) and vertically (dashed) polarized incidence.

A nontapered dielectric metasurface can yield better performances (Supplementary Fig. 1). We optimize a nontapered metasurface that has $|t_s| = |t_p| = 1$ and $\phi = \pi$ at 800 nm (Supplementary Fig. 1a). At a small incident angle, the nontapered metasurface has the high transmittance and the phase difference ϕ of π at 800 nm simultaneously, which is the best condition for the large SHEL with high efficiency. (The sharp enhancement of the shift near $\theta_i = 17°$ (Supplementary Fig. 1f) originates from the vanishing $|t_p|$ and entails the exceedingly low efficiency.)

Supplementary Note 2. The effect of the taperness in the SHEL

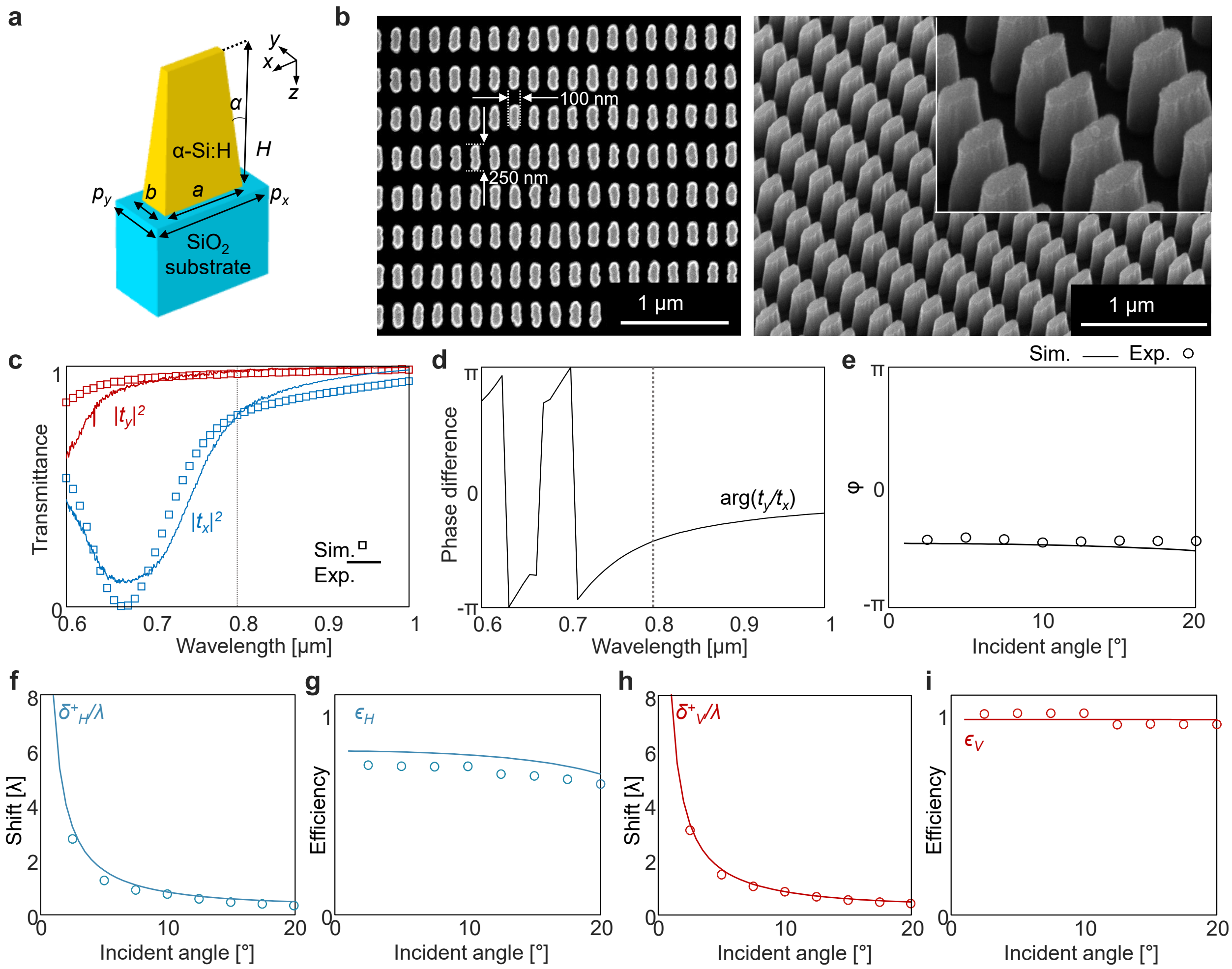

Supplementary Figure 2. Simulation and measured results of the tapered metasurface. (a) Schematic of a unit cell. A taper angle of $\alpha = 5°$ is introduced; other geometrical parameters are the same as those in Supplementary Fig. 1. (b) Scanning electron microscopy images of the fabricated sample. (c) Simulated (markers) and measured (solid) transmittance and (d) simulated ϕ under normal incidence. (e-i) Simulated (solid) and measured (markers) (e) ϕ, (f) δ_H^+/λ, (g) ϵ_H, (h) δ_V^+/λ, and (i) ϵ_V.

However, the reactive ion etching process during fabrication causes a taper angle $\alpha = 5°$ (Supplementary Figs. 2a and b). The introduction of the taperness makes ϕ deviate from $\pm\pi$, resulting in -0.47π at 800 nm (Supplementary Figs. 2d and e). The shift measured by using the Stokes polarimetry (see main manuscript and methods therein for details) is large, up to several wavelengths with high efficiency under both horizontal and vertical polarizations (Supplementary Figs. 2f-i).

The shift and efficiency of the both tapered and nontapered metasurfaces at $\theta_i = 5°$ are summarized in Supplementary Table 1. These results indicate that reducing the taper angle by optimization of etching process can enhance the shift by more than two-folds.

	δ_H/λ	δ_V/λ	ϵ_H	ϵ_V
Tapered	1.62	1.65	0.82	0.98
Nontapered	3.82	3.47	0.79	0.96

Supplementary Table 1. Comparison of shift and efficiency of the tapered and nontapered metasurfaces at $\theta_i = 5°$.

Supplementary Note 3. Mie resonances

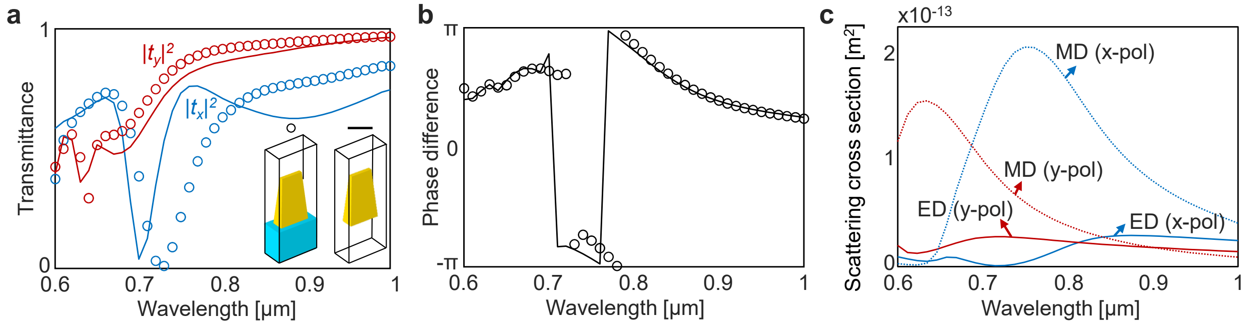

Supplementary Figure 3. Multipole expansion analysis of the metasurface (shown in Fig. 2a) under normal incidence. (a) Transmittance and (b) ϕ of the metasurface with (markers) and without (solid) substrate. (c) Multipole expansion analysis of the metasurface without substrate. ED: electric dipole, MD: magnetic dipole

The results of multipole expansion analysis of the metasurface under normal incidence are presented in Supplementary Fig. 3 for better understanding. The metasurface exhibits a strong magnetic dipole Mie resonance (Supplementary Fig. 3c, dashed), as proved by circulating displacement current density and strong magnetic field localization (Supplementary Fig. 4).

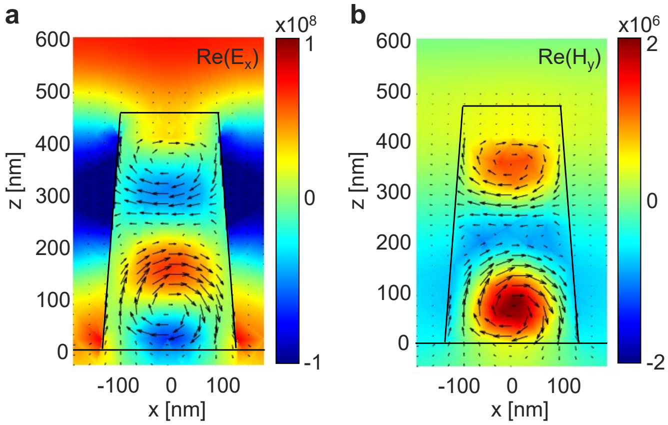

Supplementary Figure 4. Spatial field distribution of the magnetic dipole mode. (a, b) Real part of (a) E_x and (b) H_y under x-polarized incidence at $\lambda = 726$ nm. Black arrows denote displacement current density.

Supplementary Note 4. Comparison of shift and efficiency

Reference	$\|\delta/\lambda\|$	ϵ	Wavelength	Note
Our work	$\|\delta_H/\lambda\| = 2.86$; $\|\delta_V/\lambda\| = 2.40$	$\epsilon_H = 0.51$; $\epsilon_V = 0.78$	Near-infrared (800 nm)	Experimental
Ref. [1]	1.57	1	Microwave (10-20 GHz)	Experimental
Ref. [2]	$\approx$ 11.8 at 532 nm; $\approx$ 9.6 at 633 nm	$\approx 1.2\times10^{-2}$ at 532 nm; $\approx 2\times10^{-3}$ at 633 nm	Visible (532 nm, 633 nm)	Experimental
Ref. [3]	0.38 at 532 nm; 1.60 at 638 nm	0.44 at 532 nm; 0.25 at 638 nm	Visible (532 nm, 638 nm)	Experimental
Ref. [4]	3.06	1.48×10^{-4}	Visible and near-infrared (500-800 nm)	Numerical
Ref. [5]	1.43	1	NA	Theoretical (not feasible)
Ref. [6]	$\|\delta_H/\lambda\| = 7.23$; $\|\delta_V/\lambda\| = 0.11$	$\epsilon_H = 2.86\times10^{-5}$; $\epsilon_V = 0.13$	Visible (633 nm)	Experimental

Supplementary Table 2. Comparison of our work to previous studies on SHEL. The shifts and efficiencies in our work are evaluated at $\theta_i = 6°$ and at 800 nm where the experiments were performed. Ref. [4] and ref. [5] present maximum values in the given parameter space. Ref. [2] and ref. [3] show results at $\theta_i = 6°$ and at two wavelengths at which the experiments were performed. The shifts and efficiencies in ref. [6] are evaluated at $0.99\theta_B$ for H-polarization and at θ_B for V-polarization. The slight deviation from θ_B is introduced to avoid a singularity of shift at θ_B.

The shifts and efficiencies in our work and previous work [1–6] are compared in Supplementary Table 2. Besides the lossless ENZ material [5], which is not feasible, there has been only one previous demonstration that enhances the shift and efficiency simultaneously, but its operating regime is restricted to the microwave [1]. Other studies in the visible exhibit lower efficiencies. It clearly proves that our work is the first demonstration of efficient and large SHEL in the optical wavelengths.

Supplementary Note 5. A reflective metasurface for the large SHEL with high efficiency

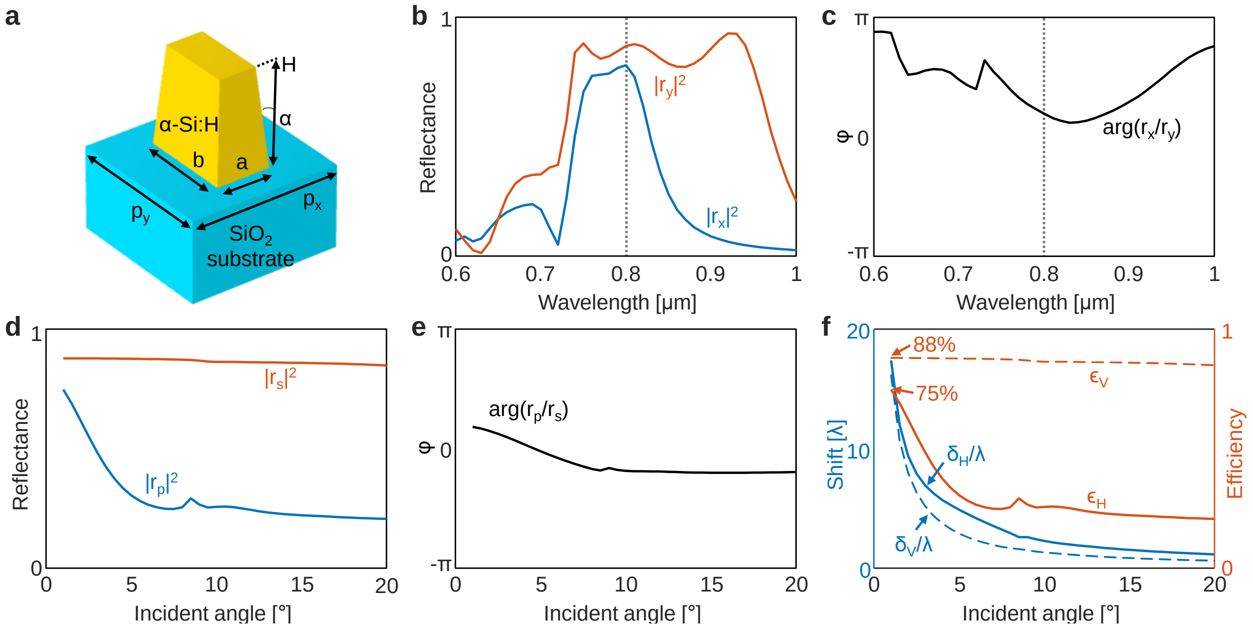

Supplementary Figure 5. A reflective metasurface for the large SHEL with high efficiency. (a) Schematic of a unit cell. Geometric parameters are given as H = 320 nm, a = 180 nm, b = 300 nm, p_x = 500 nm, and p_y = 500 nm. (b, c) Simulated (b) reflectance and (c) ϕ under normal incidence. (d) Reflectance and (e) ϕ under oblique incidence. (f) The shift and efficiency under the horizontally (solid) and vertically (dashed) polarized incidence.

The large SHEL with high efficiency can be also achieved in reflection type by exploiting a dielectric metasurface with different dimensions (Supplementary Fig. 5).

Supplementary Note 6. Weak measurement

The main manuscript contains experimental results obtained by the Stokes polarimetry setup. For completeness, this section provides a shift experimentally measured using the weak measurement. The setup parameters and results of the weak measurements are summarized in Supplementary Table 3.

	Value
θ_i	$10°$
λ	800 nm
Input pol.	Vertical
β	$3.5°$
Weak value	5.9×10^{-5} m
A	56.25
δ	1.31λ

Supplementary Table 3. Setup parameters and results of the weak measurement. β is the relative angle between the postselection polarizer and the orthogonal axis with respect to the preselection polarizer, i.e., the postselection state is $\left(\cos\beta, \quad \sin\beta\right)$, and A is an amplification factor.

The shift measured using the weak measurement technique agrees well with the results from the Stokes polarimetry setup as shown in Supplementary Fig. 6.

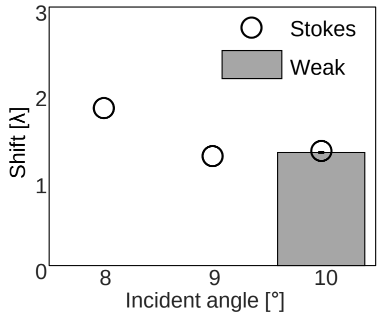

Supplementary Figure 6. Experimentally measured shifts.

Note that the effect of the shift at the back surface of the substrate is considered as $\delta_{\pm}^{V} = \pm(\delta_{sub}^{V} + \delta^{V}\frac{t_p}{t_s})$, where $\delta_{\pm}^{V}$ is the experimentally measured shift, δ_{sub}^{V} is the shift at the back surface of the substrate, δ^{V} is the shift at the metasurface, and t_p and t_s are Fresnel coefficients at the back surface [7]. The shift at the back surface is two orders of magnitude smaller than the shift at the metasurface and hence gives a negligible change.

Supplementary Note 7. Optical properties of the hydrogenated amorphous silicon

Experimentally measured permittivity of the hydrogenated amorphous silicon [8] is shown in Supplementary Fig. 7.

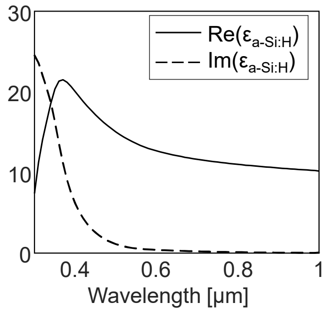

Supplementary Figure 7. Permittivity of the hydrogenated amorphous silicon. Real (solid) and imaginary (dashed) parts of permittivity of the hydrogenated amorphous silicon measured by ellipsometry.

Supplementary References

[1] M. Kim, D. Lee, H. Cho, B. Min and J. Rho. Spin Hall Effect of Light with Near-Unity Efficiency in the Microwave. *Laser Photonics Rev.* **15**(2), 2000393 (2021).

[2] O. Takayama, J. Sukham, R. Malureanu, A. V. Lavrinenko and G. Puentes. Photonic spin Hall effect in hyperbolic metamaterials at visible wavelengths. *Opt. Lett.* **43**(19), 4602–4605 (2018).

[3] M. Kim, D. Lee, T. H. Kim, Y. Yang, H. J. Park and J. Rho. Observation of enhanced optical spin Hall effect in a vertical hyperbolic metamaterial. *ACS Photonics* **6**(10), 2530–2536 (2019).

[4] M. Kim, D. Lee, B. Ko and J. Rho. Diffraction-induced enhancement of optical spin Hall effect in a dielectric grating. *APL Photonics* **5**(6), 066106 (2020).

[5] W. Zhu and W. She. Enhanced spin Hall effect of transmitted light through a thin epsilon-near-zero slab. *Opt. Lett.* **40**(13), 2961–2964 (2015).

[6] Y. Qin, Y. Li, H. He and Q. Gong. Measurement of spin Hall effect of reflected light. *Opt. Lett.* **34**(17), 2551–2553 (2009).

[7] O. Hosten and P. Kwiat. Observation of the spin Hall effect of light via weak measurements. *Science* **319**(5864), 787–790 (2008).

[8] Y. Yang, G. Yoon, S. Park, S. D. Namgung, T. Badloe, K. T. Nam and J. Rho. Revealing Structural Disorder in Hydrogenated Amorphous Silicon for a Low-Loss Photonic Platform at Visible Frequencies. *Adv. Mater.* **33**(9), 2005893 (2021).
